# Interventional effects of oral microecological agents on perioperative indicators of colorectal cancer: a meta-analysis

**DOI:** 10.3389/fonc.2023.1229177

**Published:** 2023-08-23

**Authors:** Xueyan Wang, Lijun Pan, Feiqing Wang, Fengxi Long, Bing Yang, Dongxin Tang

**Affiliations:** ^1^ The First College of Clinical Medicine, Guizhou University of Traditional Chinese Medicine, Guiyang, Guizhou, China; ^2^ Department of Medical Affairs, The First Affiliated Hospital of Guizhou University of Traditional Chinese Medicine, Guiyang, Guizhou, China; ^3^ Research Laboratory, The First Affiliated Hospital of Guizhou University of Traditional Chinese Medicine, Guiyang, Guizhou, China; ^4^ Development Planning Division, Guizhou University of Traditional Chinese Medicine, Guiyang, Guizhou, China; ^5^ Student Management Office, The First Affiliated Hospital of Guizhou University of Traditional Chinese Medicine, Guiyang, Guizhou, China; ^6^ Dean’s Office, The First Affiliated Hospital of Guizhou University of Traditional Chinese Medicine, Guiyang, Guizhou, China

**Keywords:** colorectal cancer, perioperative period, microecological agents, meta-analysis, intestinal flora

## Abstract

**Purpose:**

To investigate the efficacy of the application of microecological agents in patients with perioperative colorectal cancer.

**Methods:**

The seven electronic databases including PubMed, Cochrane Library, Excerpt Medica Database (Embase), Web of Science (WOS), Chinese Biomedical Literature Database (CBM), China National Knowledge Infrastructure (CNKI), and Wan-fang Database were systematically searched for eligible studies from 2000 to February 2023.

**Results:**

A total of 38 randomized controlled clinical trials were included in this study, with a total of 1765 patients in the microecological preparation group and 1769 patients in the control group. All data were analyzed using Review Manager 5.4 and R 4.2.2 software. Meta-analysis showed that in the perioperative period of colorectal cancer, the microecological agents group reduced patients’ adverse drug reactions, improved intestinal flora with *Lactobacillus* (SMD, 3.0858, [2.0197; 4.1520], p< 0. 0001), *Bifidobacterium* (SMD, 2.1551, [1.6145; 2.6956], p< 0.0001) and *Escherichia coli* (SMD, -1.1393, [-1.6247; -0.6538], p< 0.0001); protection of intestinal mucosal barrier function, endotoxin (SMD, -2.6850 [-4.1399; -1.2301], p=0.0003), DAO (SMD, -2.5916, [-3.4694; -1.7137], p<0.0001) and plasma D-lactate (SMD, -5.4726, [-9.8901; -1.0551], p= 0.0152), reduced inflammatory response, IL-6 (SMD, -3.1279 [-5.7706; -0.4852], p=0.0204) and CRP (SMD, -3.9698 [-7.6296; -0.3100], p=0.0335); improved the immune function of the organism, CD4+ (SMD, 1.5817 [1.0818; 2.0817], p< 0.0001), CD4+/CD8+ (SMD, 1.2938 [0.9693; 1.6183] p< 0.0001) and IgG (SMD, 1.1376 [0.2993; 1.9759] p=0.0078), improved short-term clinical efficacy, ORR (RR, 1.5105 [1.2306; 1.8541], p< 0.0001) and DCR (RR, 0.3896 [0.2620; 0.5795], p< 0.0001).

**Conclusion:**

By increasing the number of beneficial flora such as *Lactobacillus* and *Bifidobacterium* and decreasing the number of harmful flora such as *Escherichia coli*, the micro-ecological preparation group is beneficial in improving the ecological dysregulation in colorectal cancer patients receiving different treatments in the perioperative period. The microecological preparation group was able to reduce many types of adverse drug reactions, such as infections and gastrointestinal discomfort, compared to the control group. The microecological agents also reduced inflammatory responses, decreased the increase in harmful metabolites, enhanced patients’ immune function, protected intestinal mucosal barrier function, and improved short-term clinical outcomes.

**Systematic review registration:**

https://inplasy.com/inplasy-2023-4-0051/, identifier INPLASY202340051.

## 1 Introduction

Colorectal cancer(CRC) is one of the top three causes of cancer deaths worldwide, and the number of cases and deaths are on the rise, and the incidence rate among young people (20-49 years old) has increased significantly, with CRC ranking third in incidence rate and second in mortality rate in 2020 (1, 2).

Microorganisms play a crucial role in human health and disease development, colonizing various parts of our body (3–5), and having different types of crosstalk with various organs, but the highest numbers are found in the intestine (6). Gut microbes interact with the immune system, providing signals to promote the maturation of immune cells and the normal development of immune function (7, 8), which in turn is a major force in the regulation of cancer. Studies have shown that the occurrence of CRC is closely related to disorders of the intestinal microbiota (9).

CRC patients have significant ecological dysbiosis in their intestinal flora, and the various treatments that CRC patients receive during the perioperative period can cause changes in intestinal flora, and intestinal flora disorders can cause a series of adverse effects including increased intestinal inflammatory responses and harmful metabolites. In addition to preoperative mechanical bowel preparation, chemotherapy, radiotherapy, antibiotics and acid suppressants, CRC surgery itself and the stress response to surgery may also affect the intestinal flora and cause significant changes in the intestinal flora structure, which may affect postoperative recovery, short-term complications and long-term oncologic outcomes (10). In recent years, microecological preparations have been successfully used to improve the intestinal microbiota for the treatment of CRC and to mitigate treatment-mediated side effects (11). A large number of probiotic bacteria, their metabolites and other prebiotic components have been shown to influence CRC incidence and mediate intestinal immunity, while they also exhibit anti-inflammatory properties (12, 13). Gut microbial metabolites, which are very important regulators of the interaction between the gut microbiota and the host immune system (14), are abundant and include short chain fatty acids (SCFAs), tryptophan metabolites, vitamins and bile acids. These metabolites have different functions, e.g. *Clostridium difficile bacteria*, *Bifidobacterium bifidum*, *Streptococcus* and *Lactobacillus* in the gut produce SCFAs that can modulate intestinal immune function by binding to G protein-coupled receptors (GPCRs), and inhibiting the activity of histone deacetylases (HDACs) (15). However, bound bile acids, such as glycochenodeoxycholic acid and glycodeoxycholic acid, promotes tumorigenesis by stimulating cancer cell growth and increasing IL-6 expression (16, 17). And oral microecological agents, not only targeting systemic immunity, are also adept at managing mucosal immunity, thus addressing the inability of systemic immunity to affect the mucosal layer in the colon (18, 19). Microecological preparation is a general term for a class of cultures (live bacteria, dead bacteria or their metabolites) that can effectively participate in the establishment of intestinal micro-ecological balance, promote the growth of normal flora and inhibit the proliferation of pathogens after ingestion by animals, which can improve the health status and growth performance of the organism (20). According to their material composition and mode of action, micro-ecological agents can be divided into three categories: probiotics, prebiotics and synbiotics (21).

In this study, we conducted a systematic evaluation and meta-analysis of intestinal flora alterations, intestinal mucosal barrier-related factors, immune function-related indices, inflammatory factors, clinical efficacy and adverse effects produced after intervention with microecological agents in the perioperative period of CRC to provide a basis for the involvement of microecological agents in the perioperative treatment of CRC.

## 2 Methods

The protocol for this systematic review was registered on INPLASY (unique ID number) and is available in full at inpla sy.com (https://inplasy.com/inplasy-2023-4-0051/).

### 2.1 Eligibility criteria and outcome measures

According to the PICOS acronym (22), the inclusion criteria were as follows:

Participants (P): ① All cases included in the study must have pathologically confirmed CRC, and no metastases to the liver or other sites ② No microecological agents, antibiotics or laxatives within 1 month prior to surgery, have an indication for surgery and undergo radical CRC surgery ③ Approved by the hospital ethics committee, the patient and family understand and are informed, voluntarily participate in this study and sign the informed consent form ④ No restrictions by gender, race or country were found.

Intervention(I): Randomized controlled clinical trial of oral microecological preparations in the perioperative period for colorectal cancer and the content of the microecological preparations is not limited.

Comparison(C): On the basis of the control group, patients in the test group received oral microecological preparations.

Outcomes(O): Clinical efficacy and safety of microecological agents.

Study design(S): Randomized controlled clinical trials.

Exclusion criteria (1): non-randomized controlled trials (2) unclear dose and periodicity of microecological agents (3) incomplete test results (4) lack of sufficient data.

The primary outcome included two efficacy measures: (I) changes in intestinal flora: mainly involving changes in the numbers of Lactobacillus, Bifidobacterium, Escherichia coli, and Enterococcus faecalis; and (II) adverse drug reactions, assessed by detecting hematologic toxicity (leukopenia), gastrointestinal reactions (nausea, vomiting, diarrhea, flatulence), infections (pulmonary, abdominal, urinary, intestinal, incisional), and anastomotic fistulas.

Secondary outcome indicators included four efficacy measures: (i) short-term clinical efficacy ORR, DCR, short-term clinical efficacy according to the World Health Organization (WHO) criteria and Response Evaluation Criteria in Solid Tumors (RECIST), short-term tumor remission including complete remission (CR), partial remission (PR), stable disease remission (SD), progressive disease remission (PD), ORR, disease control rate ORR was defined as the sum of CR and PR, and DCR was the sum of CR, PR, and SD; (ii) immune function indicators CD4+,CD8+,CD4+/CD8+, and IgA, IgG; (iii) intestinal mucosal barrier detection indicators endotoxin, Diamine oxidase (DAO), plasma D-lactate; (iv) inflammatory factors IL-6, TNF-α, CRP.

### 2.2 Search strategy and study selection

Literature search in both international (Cochrane Library, PubMed, EMBASE, and Web of Science) and Chinese (CBM, CNKI, and Wan-fang Database) databases will be systematically searched for eligible studies from 2000 to February 2023, were independently conducted by two researchers. First, the MeSH database was searched by entering Colorectal Cancer、Intestinal flora、Randomized controlled clinical trials in turn, and then by searching for the terms ((Colorectal Neoplasm or Colorectal Tumor or Colorectal Cancer or Colorectal Carcinoma) AND (Intestinal flora or Gastrointestinal Microbiome or Gut Microbiota or Gastrointestinal Microbial Community or Intestinal Microbiome)) AND (Controlled Clinical Trials, Randomized or Randomized controlled trials or Clinical Studies) or (((Colorectal Neoplasm or Colorectal Tumor or Colorectal Cancer or Colorectal Carcinoma) AND (Intestinal flora or Gastrointestinal Microbiome or Gut Microbiota or Gastrointestinal Microbial Community or Intestinal Microbiome)) AND (Controlled Clinical Trials, Randomized or Randomized controlled trials or Clinical Studies)) AND (Probiotic or Probiotics) for screening. Two investigators independently screened titles and abstracts and then read the full text of the relevant literature to confirm inclusion, and any discrepancies were discussed with a third investigator.

### 2.3 Data extraction

The following study and participant characteristics were extracted for this study, including first author, year of publication, study type, sample size, mean age of participants, drug type, drug intervention dose and duration, and outcome indicators. Any disagreements were resolved by consensus.

### 2.4 Quality assessment and evidence level

The quality of the studies was assessed by the Cochrane risk of bias tool Review Manager 5.4. Included studies were assessed at three levels, including low, unclear, and high risk of bias. The review criteria covered seven areas, including random sequence generation, allocation concealment, blinding of investigators sequence generation, allocation concealment, blinding of participants and staff, blinding of participants and staff for outcome assessment, blinding for outcome assessment, incomplete outcome data, selective reporting, and other sources of bias. Sources of bias.

### 2.5 Statistical analyses

Statistical analyses were performed using Review Manager 5.4 and R 4.2.2 software. The outcomes were mainly represented by risk ratio (RR) and standardized mean difference (SMD) with its 95% CIs. Two- tailed p< 0.05 was considered to be statistically significant. Cochrane’s Q test and I^2^ statistics were used to assess heterogeneity between studies; p ≤ 0.1 or I^2^ > 50% indicated the presence of statistical heterogeneity, and a random-effects model was used to calculate the results when statistical heterogeneity was not present, and a fixed-effects model (common effects model) was used when statistical heterogeneity was not present. Publication bias was tested using funnel plot tests when more than 10 studies reported the same results. Sensitivity analyses were performed by removing one study at a time from the pooled analysis to explore the effect of individual studies on the pooled results. Subgroup analysis was performed according to whether or not combined chemotherapy was administered.

## 3 Results

### 3.1 Literature search and study characteristics

**Figure 1:**
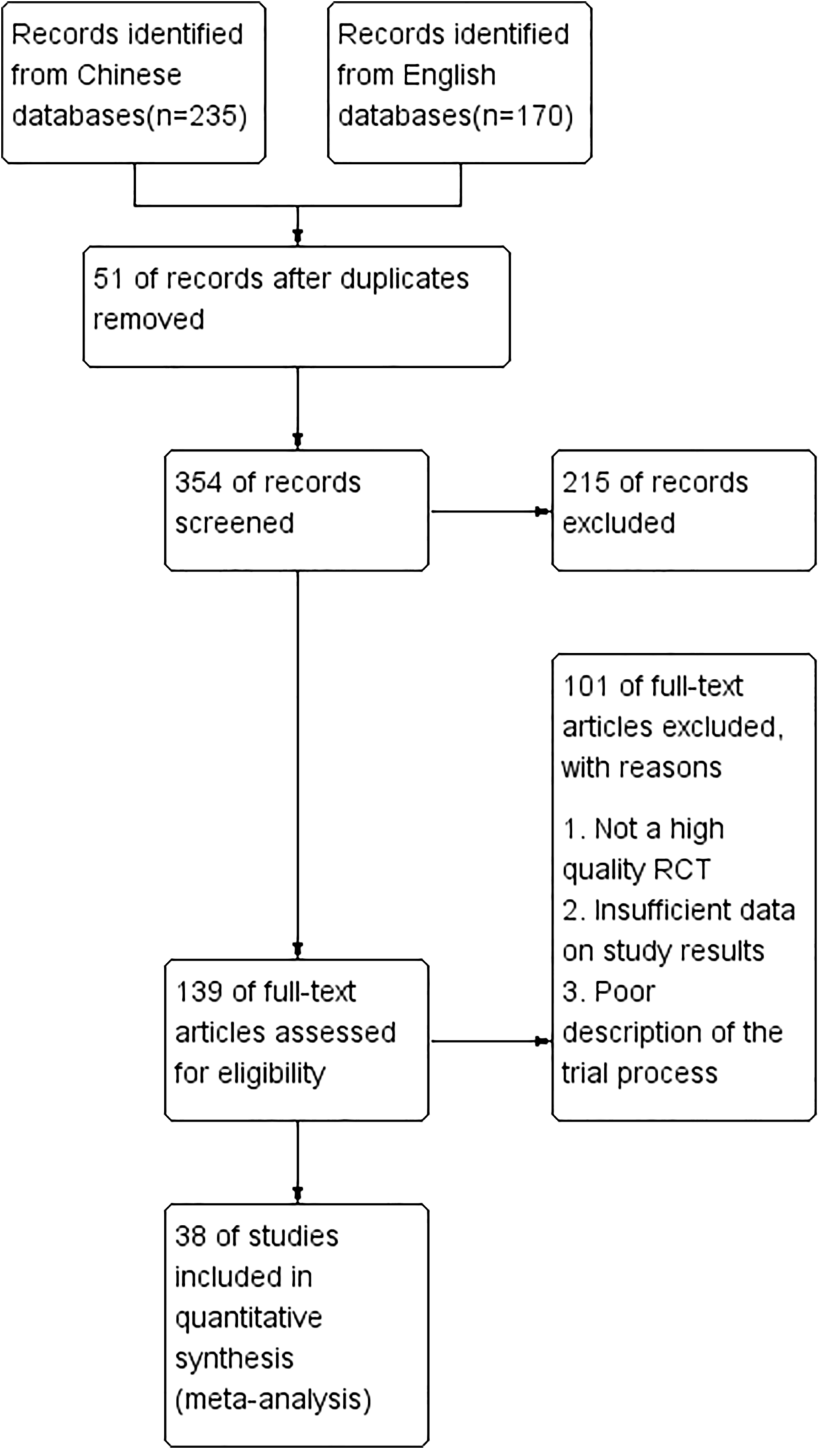
The flow charts of included studies.

A total of 405 papers were initially retrieved, and after screening titles and abstracts, 139 papers were entered for full-text reading, and 38 studies with a total of 1765 patients in the microecological preparation group and 1769 in the control group were finally included for meta-analysis (**Figure 1**). 38 studies were randomized controlled clinical studies, and their characteristics are shown in **Table S1**.

### 3.2 Methodological bias of the included studies

The method of random assignment was clearly described in all 38 studies, suggesting that there was no selection bias in all included samples. Blinding of investigators and subjects was explicitly mentioned l in some studies and not specifically described in others, suggesting possible implementation bias and measurement bias. All data were complete and did not appear to be selectively reported. Other biases are unclear, and the characteristics and quality of all included studies are shown in **Figure S1**.

### 3.3 Intestinal flora

30 (23–52) reported alterations in intestinal flora (**Table 1**; **Figures 3**, **S2**). *Lactobacillus* (I^2 ^= 96.8), *Bifidobacterium* (I^2^ = 95.3), *Escherichia coli* (I^2^ = 94.4) and *Enterococcus faecalis* (I^2^ = 98.1) were statistically heterogeneous, so the random- effects model was used for all four data. Meta-analysis showed that the microecological agents in the *Lactobacillus* (SMD, 3.0858, [2.0197; 4.1520], p< 0.0001) in the microecological preparation group, both in combination with chemotherapy (SMD, 3.09, [2.02; 4.15], p< 0.0001) and without chemotherapy (SMD, 3.75, [1.78; 5.73], p< 0.0001), improved better than the control group. The same conclusion was found for *Bifidobacterium* (SMD, 2.1551, [1.6145; 2.6956], p< 0.0001) and *E. coli* (SMD, -1.1393, [-1.6247; -0.6538], p< 0.0001). No statistically significant results were found for both groups in *Enterococcus faecalis* (SMD, -0.7515, [-1.6823; 0.1792], p=0.1135>0.05).

**Table 1:** Results of meta-analysis of intestinal flora.

Outcomes	Trials	SM	SMD,95% CI	I^2^ (%)	Q	p	PB
***Lactobacillus***	28	REM	3.0858, [2.0197; 4.1520]	96.8	< 0.0001	< 0.0001	Yes
***Bifidobacterium***	30	REM	2.1551, [1.6145; 2.6956]	95.3	< 0.0001	< 0.0001	Yes
***Escherichia coli***	29	REM	-1.1393, [-1.6247; -0.6538]	94.4	< 0.0001	< 0.0001	Yes
***Enterococcus faecalis***	24	REM	-0.7515, [-1.6823; 0.1792]	98.1	< 0.0001	0.1135	Yes

**Figure 2:**
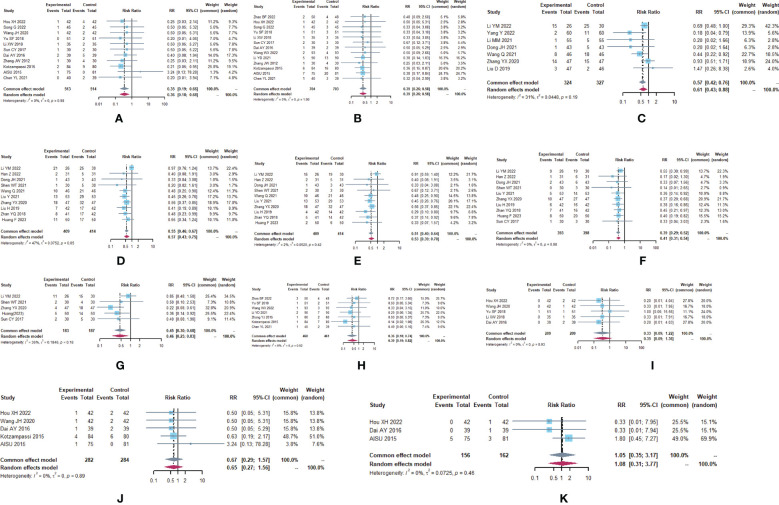
Adverse drug reactions **(A)** Forest plot of lung infection analysis results. **(B)** Forest plot of incision infection analysis results. **(C)** Forest plot of leukopenia analysis results. **(D)** Forest plot of nausea analysis results. **(E)** Forest plot of vomiting analysis results. **(F)** Forest plot of diarrhea analysis results. **(G)** Forest plot of the results of the analysis of gastrointestinal distension. **(H)** Forest plot of the results of anastomotic fistula analysis. **(I)** Forest plot of the results of the analysis of abdominal infections. **(J)** Forest plot of urinary tract infection analysis results. **(K)** Forest plot of the results of the analysis of intestinal infections.

Forest of all results are in **Figure 2**.

CI, confidence interval; PB, Publication bias; SM, statistical method; SMD, Standardized mean difference.

**Figure 3:**
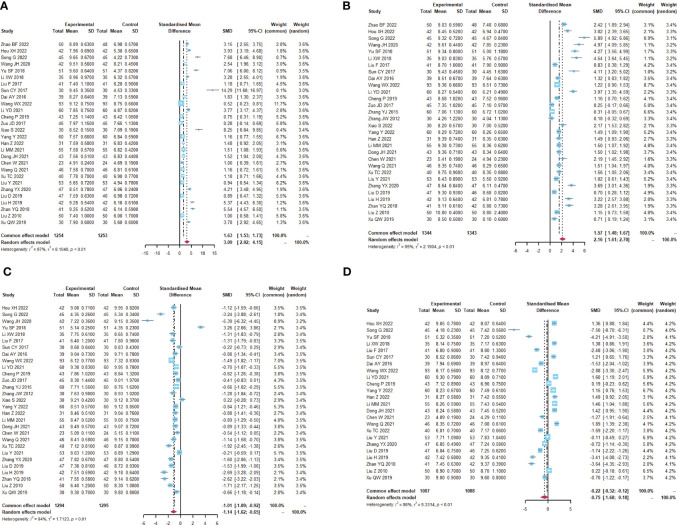
Altered intestinal flora. **(A)** Forest plot of the results of *Lactobacillus* analysis. **(B)** Forest plot of the results of *Bifidobacterium* analysis. **(C)** Forest plot of the results of *E. coli* analysis. **(D)** Forest plot of the results of the analysis of *Enterococcus faecalis*.

### 3.4 Adverse drug reactions

A total of 29 trials (24–28, 30–32, 34–39, 41–45, 47, 49–51, 53–58) reported adverse drug reactions (**Table 2**; **Figure 2**), with moderate heterogeneity in nausea (I^2^ = 47.1). The results showed that compared to the control group, the microecological agent group showed pulmonary infection (RR, 0.3499, [0.1865; 0.6564], p=0.0011), incisional infection (RR, 0.3896, [0.2620; 0.5795], p<0.0001), leukocytopenia (RR, 0.5684, [0.4228; 0.7642], p=0.0002), nausea (RR, 0.5679, [0.4294; 0.7511], p<0.0001), vomiting (RR, 0.5679, [0.4294; 0.7511], p<0.0001), diarrhea (RR, 0.3895, [0.2932; 0.5174], p< 0.0001), gastrointestinal distention (RR, 0.4512, [0.2980; 0.6833], p=0.0002), and anastomotic fistula (RR, 0.3630, [0.1780; 0.7403], p=0.0053) were at low risk. The results between the two groups for abdominal infection (RR, 0.3333, [0.0914; 1.2154], p=0.0960), urinary tract infection (RR, 0.6699, [0.2863; 1.5675], p=0.3557), and intestinal infection (RR, 1.0523, [0.3494; 3.1693], p=0.9278) were not statistically significant.

**Table 2:** Results of meta-analysis of adverse reactions.

Outcomes	Trials	Microecological preparation group	Control group	SM	RR,95% CI	I^2^ (%)	Q	p	PB
		(Events/Total)	(Events/Total)						
**Lung infection**	11	11/513	34/514	CEM	0.3499, [0.1865; 0.6564]	0.0	0.9776	0.0011	No
**Incisional infection**	13	31/704	80/703	CEM	0.3896, [0.2620; 0.5795]	0.0	1.0000	<0.0001	No
**Abdominal infection**	5	1/209	7/209	CEM	0.3333, [0.0914; 1.2154]	0.0	0.9344	0.0960	No
**Urinary tract infection**	5	8/282	12/284	CEM	0.6699, [0.2863; 1.5675]	0.0	0.8911	0.3557	No
**Intestinal infection**	3	5/156	5/162	CEM	1.0523, [0.3494; 3.1693]	0.0	0.4571	0.9278	No
**Anastomotic fistula**	7	10/468	27/461	CEM	0.3630, [0.1780; 0.7403]	0.0	0.9218	0.0053	No
**Lowered white blood cells**	7	44/324	81/327	CEM	0.5684, [0.4228; 0.7642]	30.9	0.1922	0.0002	No
**Nausea**	10	92/409	171/414	REM	0.5679, [0.4294; 0.7511]	47.1	0.0485	<0.0001	Unclear
**Vomiting**	10	72/409	146/414	CEM	0.5062, [0.4033; 0.6353]	2.5	0.4163	<0.0001	No
**Diarrhea**	10	49/393	130/398	CEM	0.3895, [0.2932; 0.5174]	0.0	0.9802	<0.0001	No
**Gastrointestinal distention**	5	24/183	56/187	CEM	0.4512, [0.2980; 0.6833]	35.8	0.1828	0.0002	No

**Figure 4:**
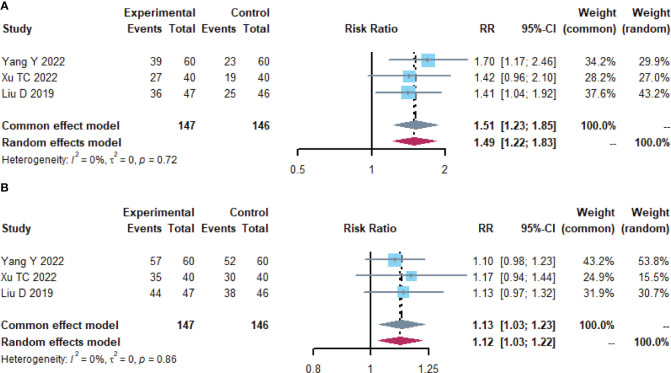
Short-term clinical efficacy. **(A)** Forest plot of ORR analysis results. **(B)** Forest plot of DCR analysis results.

Forest of all results are in **Figure 4**.

CI, confidence interval; CEM, common effects model; PB, Publication bias; REM, random- effects model; RR, relative ratio; SM, statistical method.

### 3.5 Short-term clinical efficacy

A total of 293 subjects from 3 trials (26, 29, 39)reported short-term clinical efficacy (**Table 3**; **Figure 4**). meta-analysis showed no heterogeneity in ORR and DCR results (I^2^ = 0). Compared with the control group, the microecological preparation group had better ORR (RR, 1.5105 [1.2306; 1.8541], p< 0.0001) and DCR (RR, 0.3896 [0.2620; 0.5795], p< 0.0001).

**Table 3:** Results of meta-analysis of Short-term clinical efficacy.

Outcomes	Trials	Microecological preparation group	Control group	SM	RR,95% CI	I^2^ (%)	Q	p	PB
		(Events/Total)	(Events/Total)						
**ORR**	3	102/147	67/146	CEM	1.5105, [1.2306; 1.8541]	0.0	0.7231	< 0.0001	No
**DCR**	3	136/147	120/146	CEM	0.3896, [0.2620; 0.5795]	0.0	1.0000	<0.0001	No

### 3.6 Intestinal mucosal barrier function

**Figure 5:**
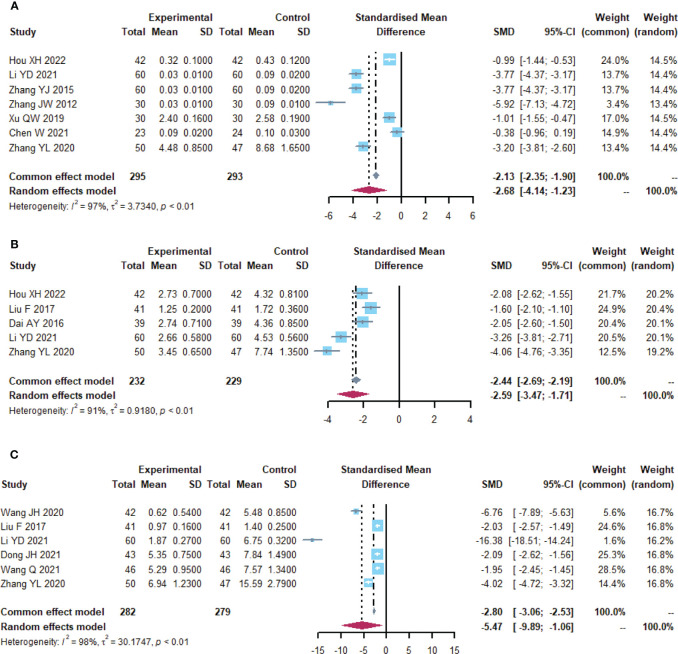
Intestinal mucosal barrier function. **(A)** Forest plot of endotoxin analysis results. **(B)** Forest plot of DAO analysis results. **(C)** Forest plot of plasma D-lactate analysis results.

A total of 12 trials (25, 32–34, 36, 37, 42, 46, 49–51, 59) reported the detection of intestinal mucosal barrier-related factors (**Table 4**; **Figures 5**, **S3**), endotoxin (I^2^ = 96.7), DAO (I^2^ = 90.7) and plasma D-lactate (I^2^ = 97.9) were statistically heterogeneous and therefore all were calculated using a random effects model. Meta-analysis results showed that compared to the control group, the microecological preparation group improved endotoxin (SMD, -2.6850[-4.1399; -1.2301], p=0.0003), DAO (SMD, -2.5916, [-3.4694; -1.7137], p<0.0001) and plasma D-lactate (SMD, -5.4726, [-9.8901; -1.0551], p= 0.0152) better.

**Table 4:** Results of meta-analysis of indicators related to intestinal mucosal barrier function.

Outcomes	Trials	SM	SMD,95% CI	I^2^ (%)	Q	p	PB
**Endotoxin**	7	REM	-2.6850, [-4.1399; -1.2301]	96.7	<0.0001	0.0003	Yes
**DAO**	5	REM	-2.5916, [-3.4694; -1.7137]	90.7	< 0.0001	< 0.0001	Yes
**Plasma D-lactate**	6	REM	-5.4726, [-9.8901; -1.0551]	97.9	< 0.0001	0.0152	Yes

### 3.7 Immune function

**Figure 6:**
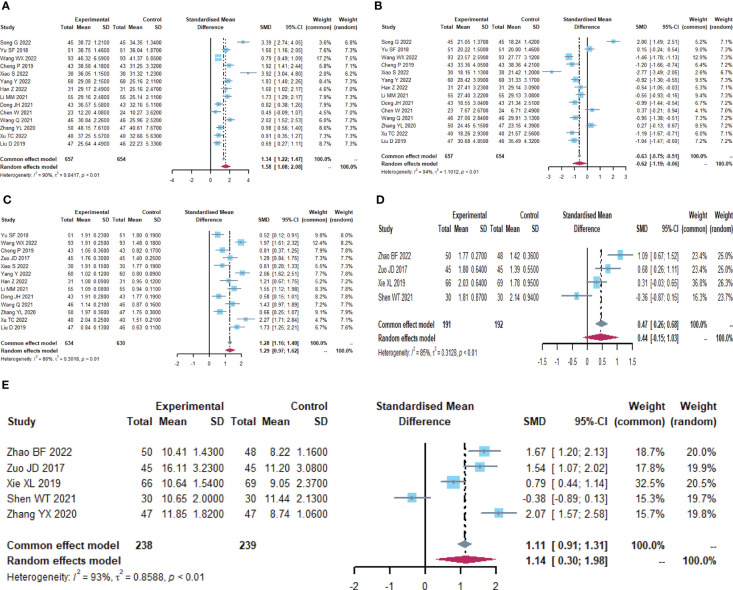
Immune function **(A)** Forest plot of CD4^+^ analysis results. **(B)** Forest plot of CD8^+^ analysis results. **(C)** Forest plot of CD4^+^/CD8^+^ analysis results. **(D)** Forest plot of IgA analysis results. **(E)** Forest plot of IgG analysis results.

A total of 19 trials (23, 24, 26–34, 38–40, 43, 48, 55, 59, 60) reported immune function-related indices (**Table 5**; **Figures 6**, **S4**), CD4+(I^2^ = 90.5), CD8+ (I^2^ = 94.3), CD4+/CD8+ (I^2^ = 85.8), IgA (I^2 ^= 85.2), IgG (I^2^ = 93.2) were statistically heterogeneous, so a random-effects model was used. Meta-analysis results showed that the microecological preparation group improved CD4+ (SMD, 1.5817 [1.0818; 2.0817], p< 0.0001) CD4+/CD8+ (SMD, 1.2938[0.9693; 1.6183] p< 0.0001) and IgG (SMD, 1.1376[0.2993; 1.9759] p=0.0078) compared to the control group. The difference between the two groups for CD8+ (SMD, -0.6248[-1.1885; -0.0611] p=0.0298) and IgA (SMD, 0.4396 [-0.1487; 1.0279], p= 0.1430) were not statistically significant.

**Table 5:** Results of meta-analysis of immune function-related indicators.

Outcomes	Trials	SM	SMD,95% CI	I^2^ (%)	Q	p	PB
**CD4^+^**	14	REM	1.5817, [1.0818; 2.0817]	90.5	< 0.0001	< 0.0001	Yes
**CD8^+^**	14	REM	-0.6248, [-1.1885; -0.0611]	94.3	< 0.0001	0.0298	Yes
**CD4^+^/CD8^+^**	13	REM	1.2938, [0.9693; 1.6183]	85.8	< 0.0001	< 0.0001	Yes
**IgA**	4	REM	0.4396, [-0.1487; 1.0279]	85.2	0.0001	0.1430	Yes
**IgG**	5	REM	1.1376, [0.2993; 1.9759]	93.2	< 0.0001	0.0078	Yes

### 3.8 Inflammatory factors

**Figure 7:**
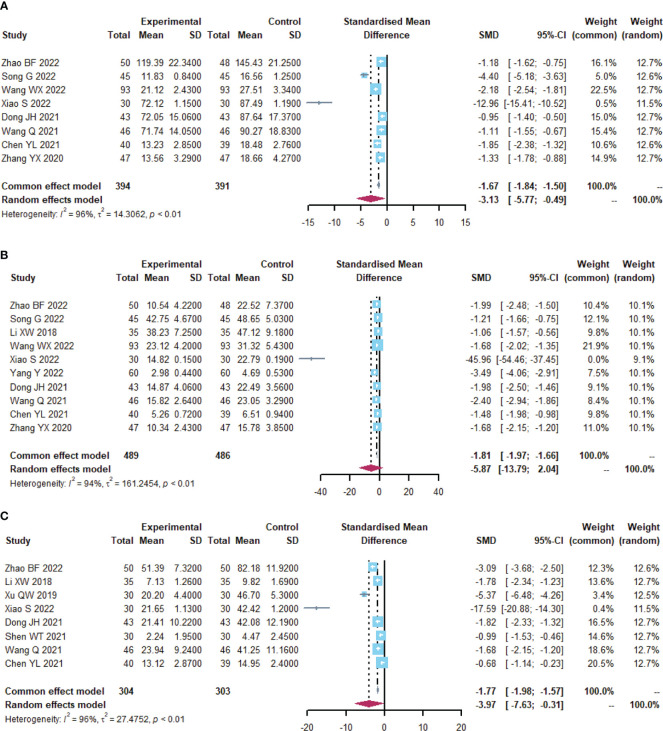
Inflammatory factors. **(A)** Forest plot of IL-6 analysis results. **(B)** Forest plot of TNF-α analysis results. **(C)** Forest plot of CRP analysis results.

A total of 12 trials (23, 24, 26, 28, 30, 32, 34, 38, 42, 44, 55, 56) reported on inflammatory factor-related indices (**Table 6**; **Figures 7**, **S5**). IL-6 (I^2^ = 95.6), TNF-α (I^2^ = 94.4), and CRP (I^2^ = 96.1) were statistically heterogeneous, so a random-effects model was chosen. IL-6 (SMD, -3.1279[-5.7706; -0.4852], p=0.0204) and CRP (SMD, -3.9698[-7.6296; -0.3100], p=0.0335) were improved in the microecological preparation group compared to the control group. no statistical difference was found in TNF-α (SMD, -5.8744[-13.7876; 2.0388], p=0.1457) between the two groups.

**Table 6:** Results of meta-analysis of indicators related to inflammatory factors.

Outcomes	Trials	SM	SMD,95% CI	I^2^ (%)	Q	p	PB
**IL-6**	8	REM	-3.1279, [-5.7706; -0.4852]	95.6	< 0.0001	0.0204	Yes
**TNF-α**	10	REM	-5.8744, [-13.7876; 2.0388]	94.4	< 0.0001	0.1457	Yes
**CRP**	8	REM	-3.9698, [-7.6296; -0.3100]	96.1	< 0.0001	0.0335	Yes

### 3.9 Publication bias analysis

**Figure 8:**
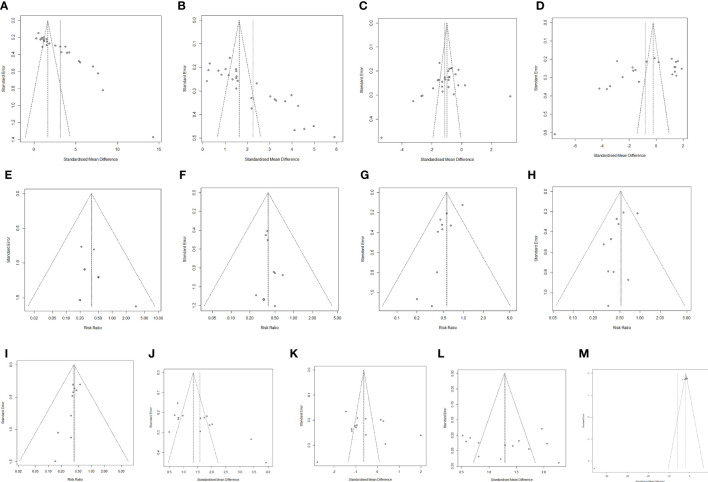
Publication bias analysis. **(A)** *Lactobacillus*. **(B)** *Bifidobacterium*. **(C)** *Escherichia coli*. **(D)** *Enterococcus faecalis*. **(E)** Lung infection **(F)** Incisional infections. **(G)** Nausea. **(H)** Vomiting. **(I)** Diarrhea. **(J)** CD4^+^. **(K)** CD8^+^. **(L)** CD4^+^/CD8^+^. **(M)** TNF-α.

Funnel plots were used to examine Lactobacillus, Bifidobacterium, Escherichia coli, Enterococcus faecalis, Lung infection, Incisional infections, Nausea, Vomiting. Diarrhea, CD4+, CD8+, CD4+/CD8+, publication bias of TNF-α (**Figure 8**).

### 3.10 Sensitivity analysis

To assess the stability of the results. The meta-analysis of the remaining literature was combined after sequentially excluding one literature, and the changes in the combined results were observed to assess whether the results of the original meta-analysis were significantly changed by certain studies (**Figure S6**).

## 4 Discussion

In recent years, a growing number of studies have shown that microecological agents can be used to treat CRC and alleviate side effects due to treatment. Meta-analysis included 38 trials containing 3,234 patients to assess whether the addition of microecological agents is beneficial in improving outcome indicators in the perioperative period of CRC.

The development of CRC is strongly associated with disturbances in the gut microbiota. The data showed that the microecological preparation group was beneficial in improving the ecological dysbiosis brought about by various treatments received by CRC patients in the perioperative period, increasing the number of beneficial flora such as *Lactobacillus* and *Bifidobacterium*, while reducing harmful flora such as *E. coli*. However, under certain conditions, it may have the opposite effect. *Bifidobacteria* may play an important role in altering host metabolism during parasitic infections, thereby promoting the development of cholangiocarcinoma (CC) (61). In intrahepatic cholangiocarcinoma (ICC), *Lactobacillus* and *Alloscardovia* were positively correlated with taurocholanol deoxycholic acid. Plasma tauroarsodeoxycholic acid was negatively correlated with Pseudomonas spp. and survival time, but positively correlated with vascular invasion (17, 62).

CRC surgery itself and the stress response to surgery can affect patients’ postoperative recovery as well as short-term complications. Compared with the control group, the microecological preparation group was able to reduce many types of adverse drug reactions such as infections and gastrointestinal discomfort. The intestinal microflora can influence the efficacy and adverse effects of chemotherapeutic drugs by regulating the body’s immune response (63, 64), regulating the body’s hormone levels (65, 66), regulating the body’s metabolic levels (67, 68), and regulating the metabolism and transport of chemotherapeutic drugs (69–72).Therefore, appropriate supplementation of probiotics, prebiotics or synbiotics by micro-ecological means is beneficial to regulate the homeostasis of the intestinal microflora and thus reduce the adverse effects of chemotherapeutic drugs.

At the same time, the microecological agents also reduced the inflammatory response, decreased the increase of harmful metabolites, enhanced the immune function of patients, and improved short-term clinical outcomes. The harmful metabolites of the gut flora, such as ammonia, phenols and p-cresol, are involved in the development and progression of cancer through chronic inflammation and DNA damage (73, 74). For example, high levels of lipopolysaccharide (LPS), entering the bloodstream can cause a number of severe pathophysiological responses, including fever, coagulation and shock, by disrupting the host’s immune, complement and coagulation systems (75). Primary bile acids enter the large intestine and are converted by intestinal bacteria into secondary bile acids, a class of metabolites with pro-cancer effects that can promote tumour development by stimulating oxidative stress (e.g. reactive oxygen species and reactive nitrogen species), inducing cellular DNA damage and activating EGFR and NF-κB (76–78).

## 5 Conclusion

The subtle interactions between the intestinal flora and human physiology can influence multiple aspects of health. Microbial-epithelial interactions can maintain intestinal barrier function, modulate resistance to infection and intestinal immune function, and maintain host metabolism.

CRC is one of the top three causes of cancer deaths worldwide, and surgery is the primary treatment for colorectal cancer. However, trauma, disturbance of normal intestinal flora, decreased intestinal mucosal barrier function, increased systemic inflammation, decreased immune function, and also the risk of postoperative infection may occur after surgery (79).

Probiotics have antitumor activity by a variety of mechanisms. The most common probiotic flora are two genera of *Lactobacilli* and *Bifidobacteria*, which are naturally present in the human digestive system. For example, antioxidants produced by *Lactobacilli* are able to fight against angiogenic factors, reduce DNA damage, reduce inflammation and tumor size, and inhibit the expression of tumor-specific proteins and polyamine components (80). In addition, prebiotics are fermentable components present in foods that alter the composition and activity of the intestinal microbiota and promote host health. One of the most commonly used prebiotics is resistant starch, which increases the biological activity of a wide range of probiotic bacteria, especially *bifidobacteria*, and modifies the immune response (81). Prebiotics are organic substances that are not digested or absorbed by the host, but can selectively promote the metabolism and proliferation of beneficial bacteria in the body, thereby improving the health of the host. Commonly used prebiotics include Fructo oligosaccharide, xylo-oligosaccharides and inulin. Studies have shown that Fructo oligosaccharide can reduce the number and activity of carcinogenic enzymes and regulate the body’s immune capacity, and the short-chain fatty acids and lactic acid it ferments to produce in the colon can reduce intestinal pH and ammonia concentration, which is conducive to the reduction and inhibition of intestinal spoilage substances (82) Xylo-oligosaccharides can inhibit the invasion of exogenous pathogenic bacteria, improve the body’s immune response and protect the barrier function of the intestinal mucosa (83).

Our study found that in the perioperative period of CRC, a more effective treatment regimen in the microecological agent group was accompanied by reduced adverse drug reactions in patients, improved intestinal flora, improved short-term clinical outcomes, enhanced body immune function, and reduced inflammatory responses. According to the World Health Organization, appropriate doses of probiotics are beneficial to human health. Proper consumption of microecological agents, such as probiotics or prebiotics, may be a promising way to prevent and treat CRC.

## Data availability statement

The original contributions presented in the study are included in the article/**Supplementary Material**. Further inquiries can be directed to the corresponding author.

## Author contributions

Conceptualization: XW. Methodology: XW. Formal analysis and investigation: XW and LP. Writing—original draft preparation: XW. Writing—review and editing: XW, FW. Funding acquisition: DT and BY. Resources: XW. Supervision: DT and FL. All authors commented on previous versions of the manuscript. All authors read and approved the final manuscript.
