# Supplementary material for: Interventional effects of oral microecological agents on perioperative indicators of colorectal cancer: a meta-analysis
Source: Front Oncol. 2023 Aug 23;13:1229177. doi: 10.3389/fonc.2023.1229177 (PMC10482437; doi:10.3389/fonc.2023.1229177)
Supplement: Supplementary file 1 [file DataSheet_1.docx]

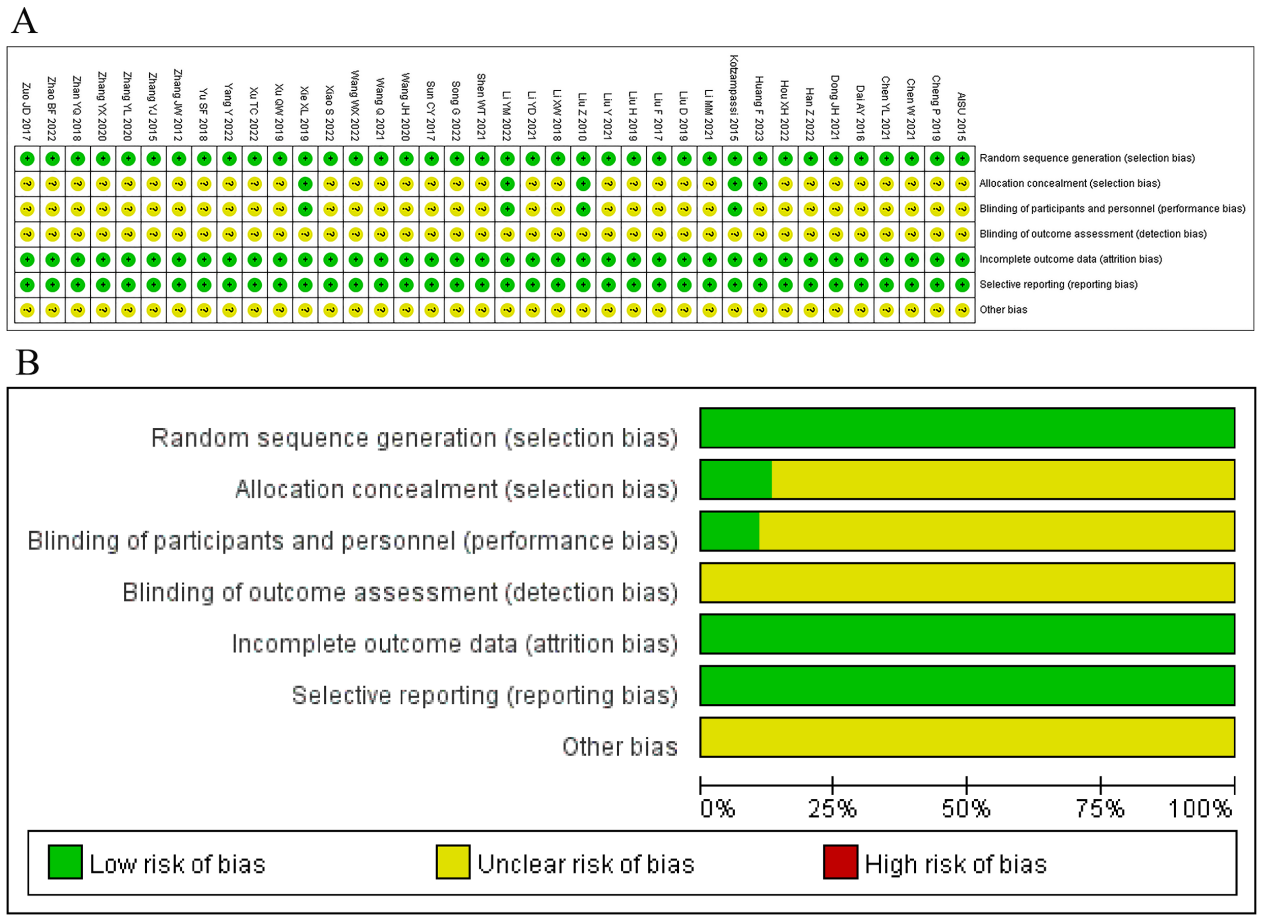


**Figure S1:** Risk of methodological bias of the included studies. (A) Risk of bias summary: review authors' judgments about each risk of bias item for each included study. (B) Risk of bias graph: review authors' judgment about each risk of bias item presented as percentages across all included studies.


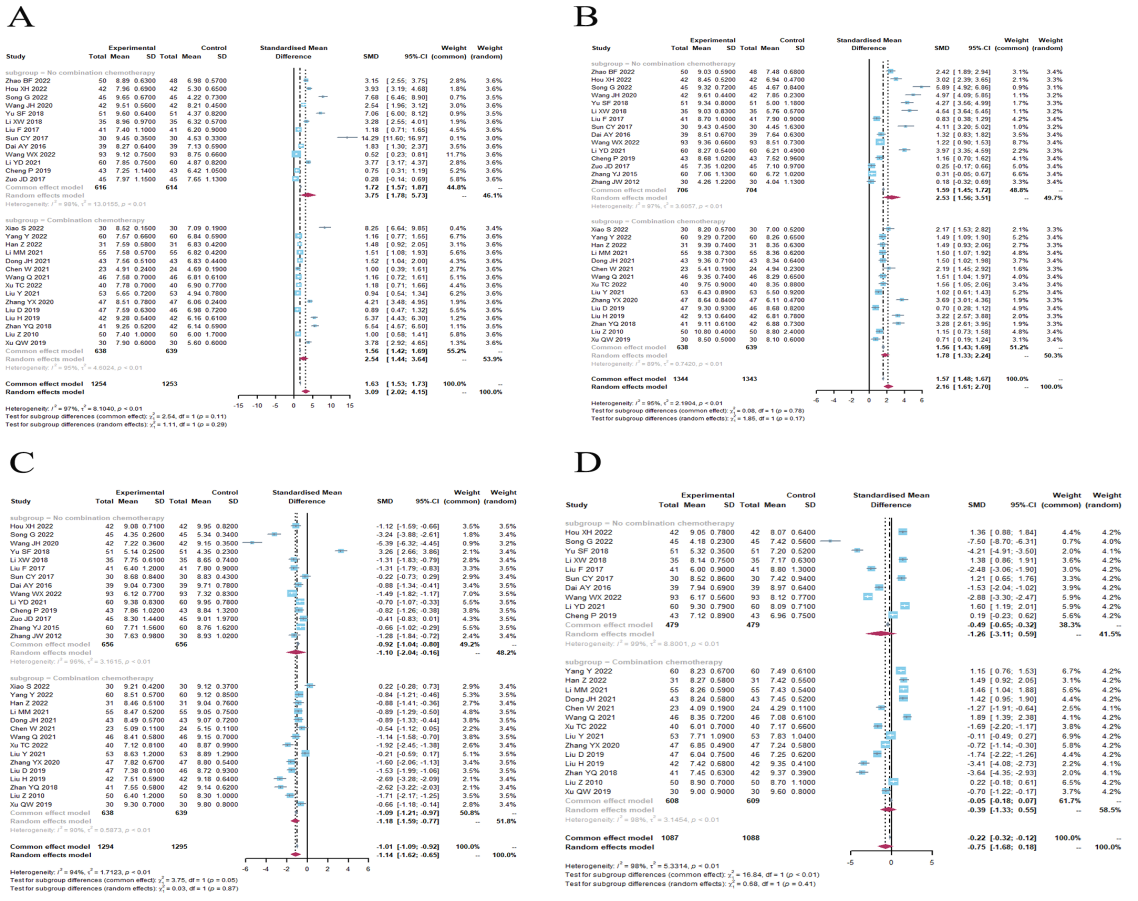


**Figure S2:** Altered intestinal flora subgroups. (A) Forest plot of the results of *Lactobacillus* subgroup analysis. (B) Forest plot of *Bifidobacterium* subgroup analysis results. (C) Forest plot of the results of *E. coli* subgroup analysis. (D) Forest plot of the results of *Enterococcus faecalis* subgroup analysis.


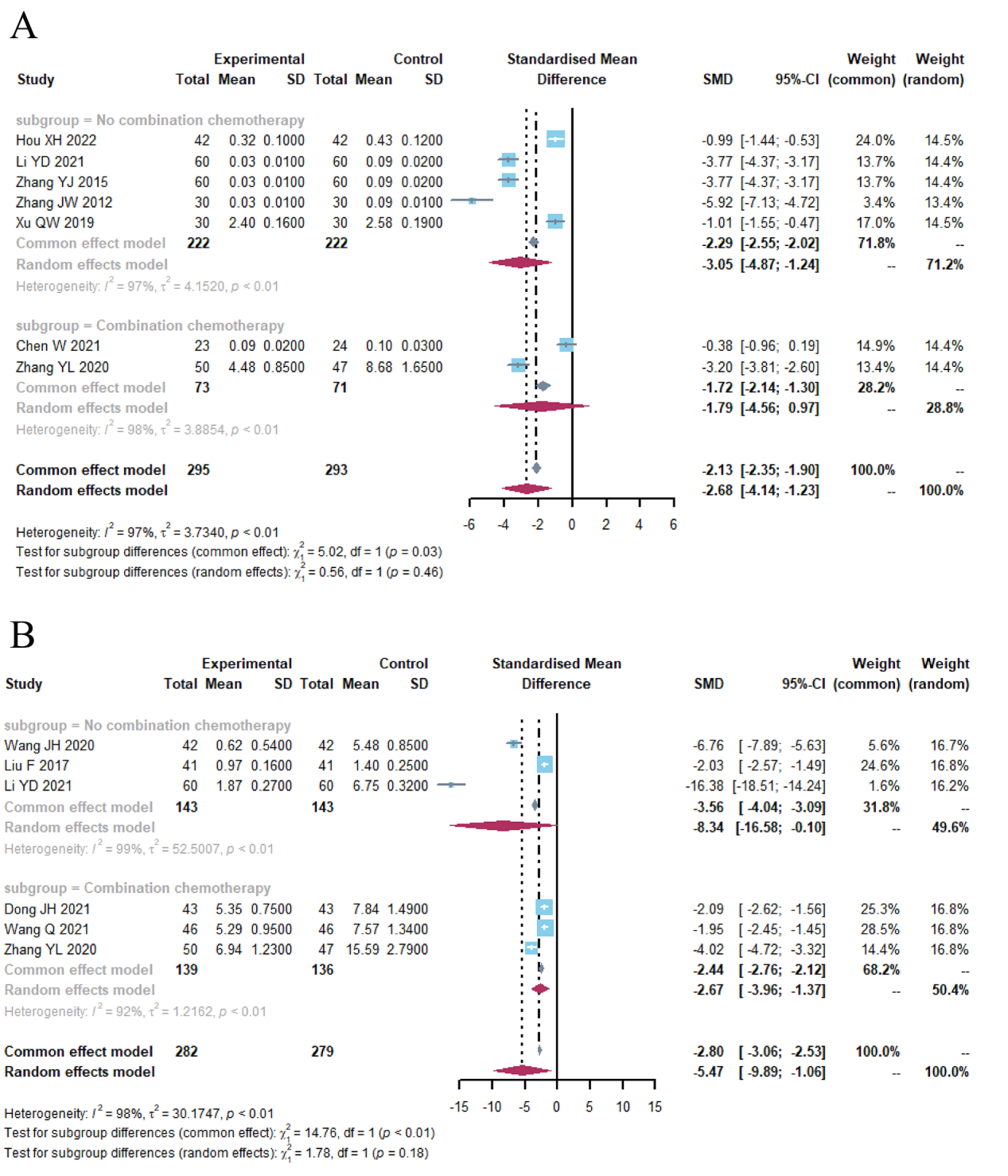


**Figure S3:** Subgroup alterations in intestinal mucosal barrier function. (A) Forest plot of the results of endotoxin subgroup analysis. (B) Forest plot of the results of plasma D-lactate subgroup analysis.


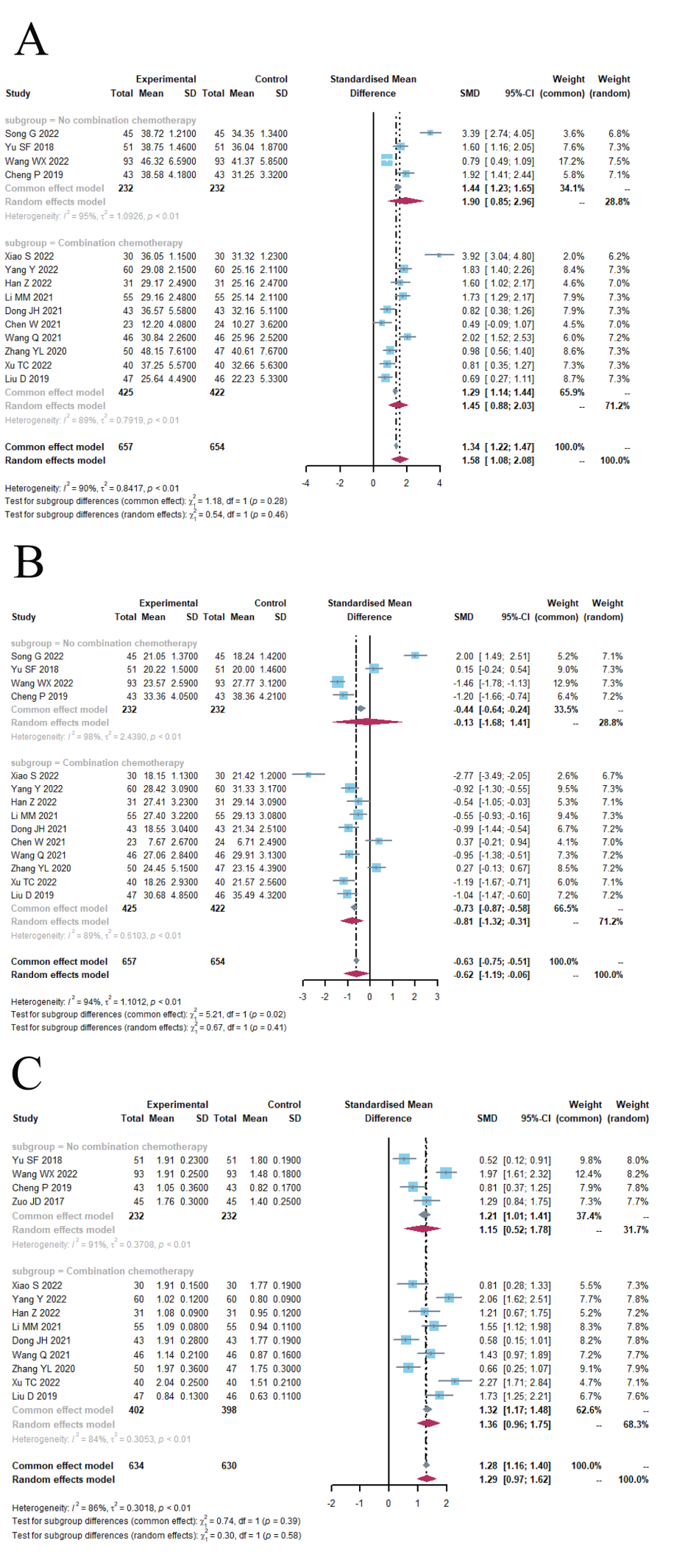


**Figure S4:** Immunological subgroups. (A) Forest plot of the results of CD4^+^ subgroup analysis. (B) Forest plot of the results of CD8^+^ subgroup analysis. (C) Forest plot of the results of CD4^+^/ CD8^+^ subgroup analysis.


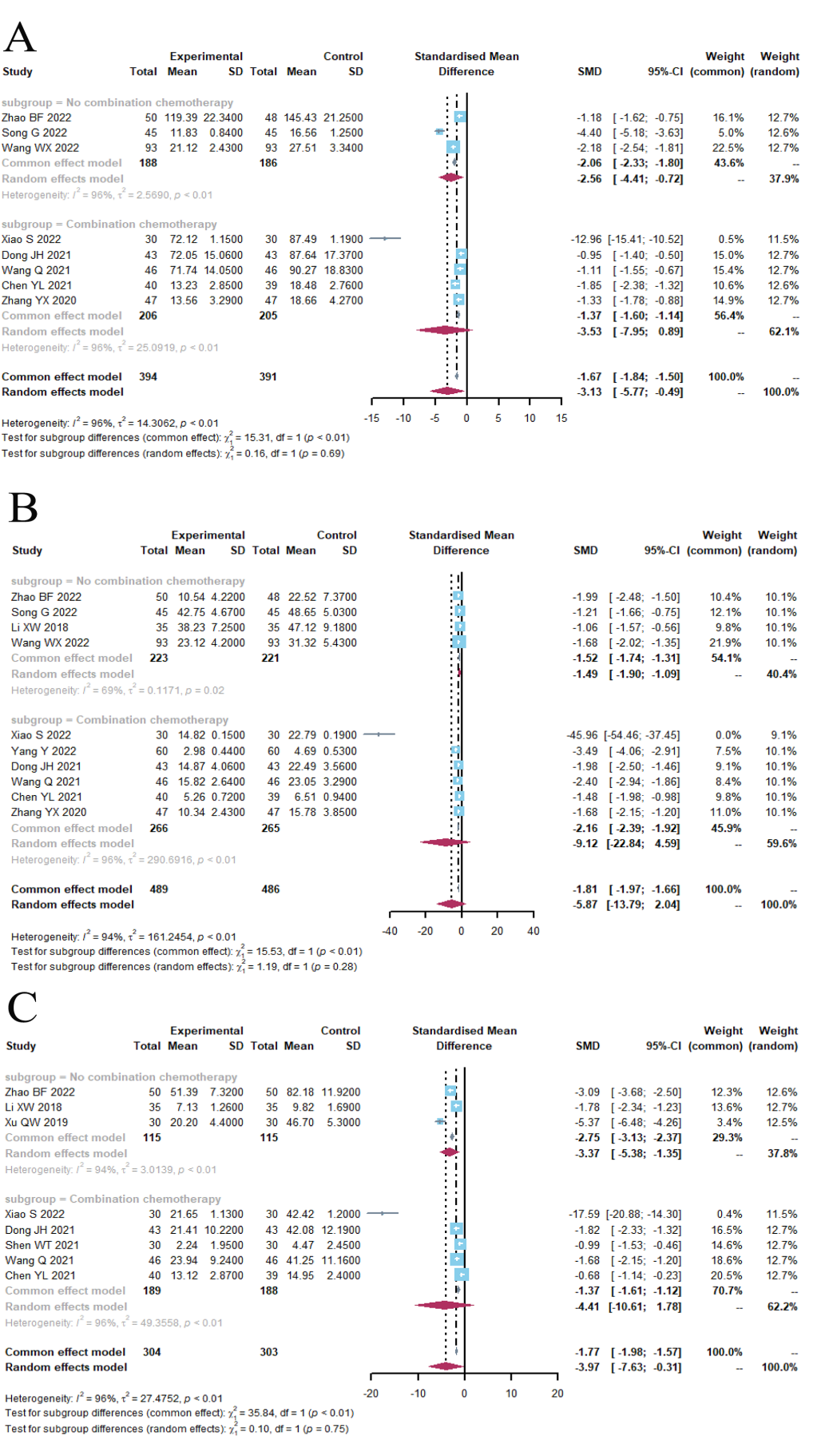


**Figure S5:** Inflammatory factor subgroup alterations. (A) Forest plot of the results of IL-6 subgroup analysis. (B) Forest plot of TNF-α subgroup analysis results. (C) Forest plot of the results of CRP subgroup analysis.


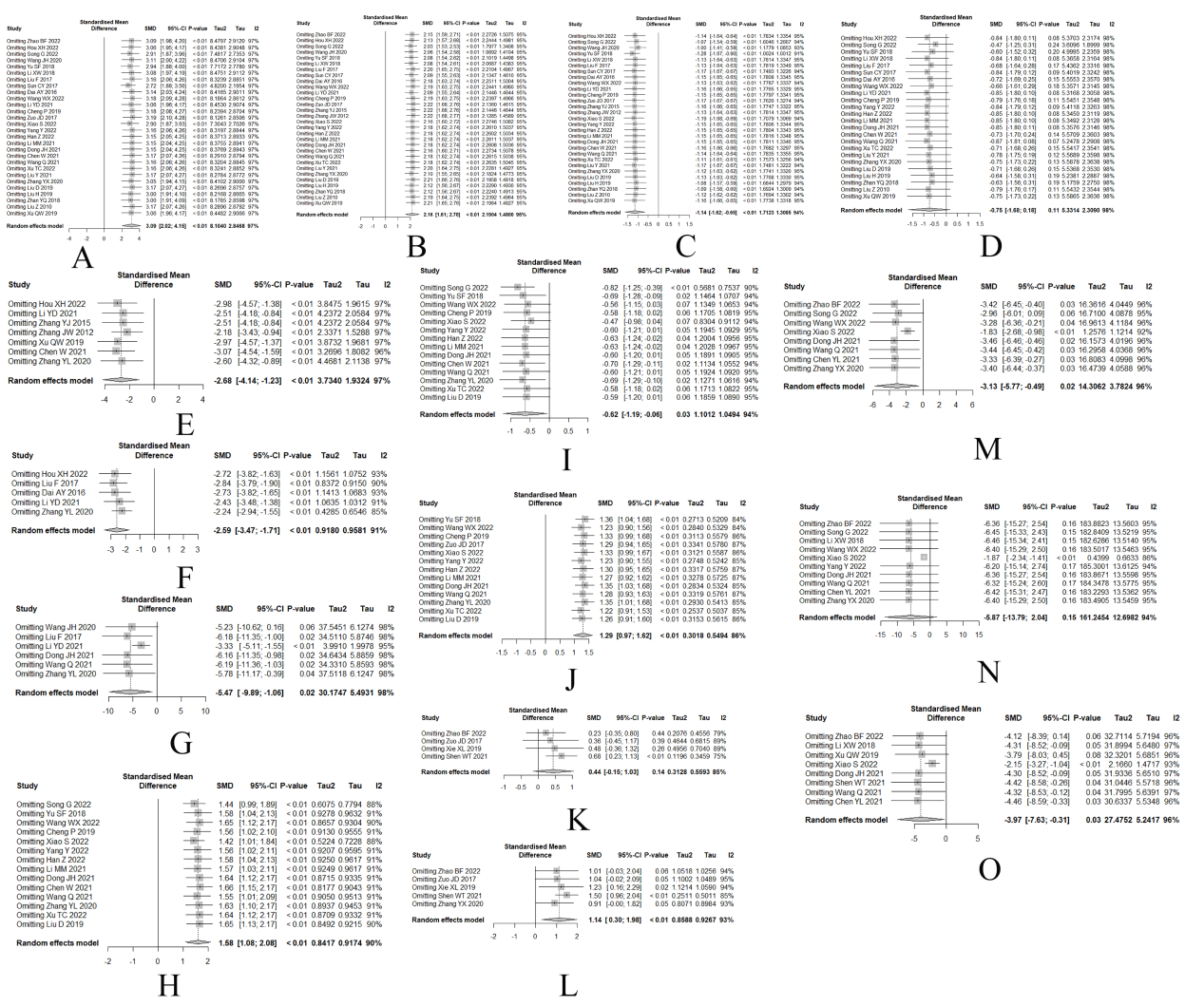


**Figure S6:** Sensitivity analysis. (A) *Lactobacillus*. (B) *Bifidobacterium*. (C) *Escherichia coli*. (D) *Enterococcus faecalis*. (E) endotoxin. (F) DAO. (G) plasma D-lactate. (H) CD4^+^. (I) CD8^+^. (J) CD4^+^/CD8^+^ (K) IgA. (L) IgG. (M)IL-6. (N) TNF-α. (O)CRP.
