# Supplementary material for: Interventional effects of oral microecological agents on perioperative indicators of colorectal cancer: a meta-analysis
Source: Front Oncol. 2023 Aug 23;13:1229177. doi: 10.3389/fonc.2023.1229177 (PMC10482437; doi:10.3389/fonc.2023.1229177)
Supplement: Supplementary file 2 [file Table_1.docx]

**Table S1:** Characteristics of randomized controlled trials of microecological preparations for intervention in the perioperative period of CRC(n=3534)

| **First author(year)** | **Design** | **Sample size**  **(M/C n)** | **Age (M/C years)** | **Sex (Male and Female n)** | | **Microecological preparation group intervention (Dosage and duration)** | **Control group intervention (Dosage and duration)** | **Outcome measures** |
| --- | --- | --- | --- | --- | --- | --- | --- | --- |
|  |  |  |  | **Microecological preparation group** | **Control group** |  |  |  |
| Huang F 2023^[53]^ | RCT | 50/50 | (57.70±11.90)/ (62.10±10.50) | 24/26 | 29/21 | Bifidobaeterium tetravaccine tablets, oral,1 capsule/time, tid, for 6weeks;  XELOX | Placebo tablets, XELOX  Ox.130 mg/m^2^, ivgtt, 3h, d1,  Xe**l** 800-1000 mg/m^2^, po, bid, d1-d14 | O7 |
| Xiao S 2022**^[23]^** | RCT | 30/30 | 43-79 (62.17±5.52)/ 44-79 (62.59±5.51) | 16/14 | 15/15 | Bifid Triple Viable Capsules, oral, 4 capsules/time, bid;  Ox. 130 mg/m^2^, ivgtt,3h  Fluorouracil Injection, 300-400 mg/m^2^, for 8weeks | Ox. 130 mg/m^2^, ivgtt,3h  Fluorouracil Injection, 300-400 mg/m^2^, for 8 weeks | O1，3，4 |
| Zhao BF 2022^[24]^ | RCT | 50/48 | 35-86(72.56±12.57)/ 43-82(71.52±12.89) | 26/24 | 22/26 | Compound probiotics, Oral, 1 packet/time, bid,5d; Routine nutritional care | Routine nutritional care | O1,3,4,7 |
| Li YM 2022^[54]^ | RCT | 26/30 | (56.78±1.73)/ (59.67±2.91) | 14/12 | 20/10 | XOS,3g/d, for 12weeks;  XELOX/ FOLFOX | XELOX/ FOLFOX, 21days/cycle, for 4 cycles  XELOX: Ox.130 mg/m^2^, ivgtt, 3h, d1, Xe**l** 800-1000 mg/m^2^, po, bid, d1-d14;  FOLFOX, Ox. 100 mg/m^2^ ivgtt,2h, d1, 5-Fu,500mg/m^2^ ivgtt d1~d5 | O1,4,6,7 |
| Hou XH 2022^[25]^ | RCT | 42/42 | 33-70(57.35±8.42)/  35-72(58.20±8.33) | 25/17 | 26/16 | Bifid Triple Viable Capsules, oral, 3 capsules/time, bid,7d; Routine nutritional care | Routine nutritional care | O1,2,7 |
| Yang Y 2022^[26]^ | RCT | 60/60 | 30-70(56.21±7.78)/  30-70(57.15±8.18) | 28/32 | 31/29 | Bifid Triple Viable Capsules, oral, 2-4 capsules/time, bid, for 24weeks; mFOLFOX6 | mFOLFOX6, 21days/cycle, for 4 cycles  Ox.85 mg/m^2^, ivgtt, 2h, d1,  5-Fu,400mg/m^2^ ivgtt, d1,  this was followed by 5-Fu, 2400 mg/m^2^ for 46h | O1,3,7 |
| Han Z 2022^[27]^ | RCT | 31/31 | 45-78（60.40±9.50）/ 48-73（60.15±10.57） | 17/14 | 18/13 | Bifid Triple Viable Capsules, oral, 3 capsules/time, tid, for 6weeks;  XELOX | XELOX,21days/cycle, for 2 cycles  Ox.130 mg/m^2^, ivgtt, 3h, d1  Xe**l** 800mg/m^2^, po, bid, d1-d14 | O1,3,7 |
| Song G 2022^[28]^ | RCT | 45/45 | 42-75(52.78±5.47)/  39-75(54.32±5.62) | 24/21 | 25/20 | Bifid Triple Viable Capsules, oral, 3 capsules/time, tid, for 10 days; Continuous subcutaneous negative pressure drainage | Continuous subcutaneous negative pressure drainage | O1,3,7 |
| Xu TC 2022^[29]^ | RCT | 40/40 | 46-69(49.86±4.47)/  45-70(49.78±4.57) | 23/17 | 22/18 | Bifid Triple Viable Capsules, oral, 3 capsules/time, tid, for 6weeks;  XELOX | XELOX, 21days/cycle, for 2 cycles  Ox.130 mg/m^2^, ivgtt, 3h, d1  Xe**l** 800mg/m^2^, po, bid, d1-d14 | O1,3,5 |
| Wang WX 2022^[30]^ | RCT | 93/93 | 33-70(51.36±7.92)/  31-71(52.19±8.33) | 51/42 | 48/45 | Bifidobaeterium tetravaccine tablets, 3 capsule/time, tid, 7d; Supportive and symptomatic management | Supportive and symptomatic management | O1,3,4,7 |
| Li MM 2021^[31]^ | RCT | 55/55 | 49-72(60.14±10.52)/ 48-69(59.26±10.37) | 35/20 | 38/17 | Bifid Triple Viable Capsules, oral, 3 capsules/time, tid, for 6weeks;  XELOX | XELOX, 21days/cycle, for 2 cycles  Ox.130 mg/m^2^, ivgtt, 3h, d1  Xe**l** 800mg/m^2^, po, bid, d1-d14 | O1,3,7 |
| Dong JH 2021^[32]^ | RCT | 43/43 | 31-58(43.64±6.55)/  30-57(42.86±6.11) | 23/20 | 26/17 | Bifid Triple Viable Capsules, oral, 4 capsules/time, bid, for 12weeks;  FLOFOX4 | FLOFOX4, 14days/cycle, for 6 cycles  Ox. 130 mg/m^2^, ivgtt, 2-4h, d1,  5-Fu,400mg/m^2^ ivgtt, d1,  this was followed by 5-Fu, 600 mg/m^2^ for 22h | O1,3,4,7 |
| Shen WT 2021^[55]^ | RCT | 30/30 | 44-77(59.93±9.29)/  24-77(56.90±10.33) | 16/14 | 19/11 | Bifidobaeterium tetravaccine tablets, 3 capsule/time, tid, for 6weeks;XELOX | XELOX, 21days/cycle, for 2 cyclesOx.130 mg/m^2^, ivgtt, 3h, d1  Xe**l** 800mg/m^2^, po, bid, d1-d14 | O3,4,7 |
| Chen W 2021^[33]^ | RCT | 23/24 | (59.13±7.87)/  (55.75±7.42) | 13/10 | 14/10 | Bifid Triple Viable Capsules, oral, 3 capsules/time, bid, 5d; CapeOX | CapeOX  Ox.130 mg/m^2^, ivgtt, 3h, d1  Cap. 1000mg/m^2^, po, bid, d1-d14 | O1,2,3,7 |
| Wang Q 2021^[34]^ | RCT | 46/46 | (43.89±8.25)/  (44.21±10.48) | 25/21 | 28/18 | Clostridium butyricum Live Tablets, 2 capsules/time, tid, for 8weeks;  FLOFOX4 | FLOFOX4, 14days/cycle, for 4 cycles  Ox. 85 mg/m^2^, ivgtt, 2h, d1,  5-Fu,400mg/m^2^ ivgtt, d1,  this was followed by 5-Fu, 600 mg/m^2^ for 22h | O1,2,3,4,6,7 |
| Chen YL 2021^[56]^ | RCT | 40/39 | 44-78(59.48±8.33)/  45-79(58.79±8.23) | 26/14 | 22/17 | Bifid Triple Viable Capsules, oral, 3 capsules/time, tid, for 4weeks;  mFOLFOX | mFOLFOX 14days/cycle, for 2 cycles  Ox. 85 mg/m^2^, ivgtt, 2h, d1,  5-Fu,400mg/m^2^ ivgtt, d1,  this was followed by 5-Fu, 2400 mg/m^2^ for 46h | O4,7 |
| Liu Y 2021^[35]^ | RCT | 53/53 | (64.10±10.90)/  (62.60±10.30) | - | - | Bifid Triple Viable Capsules, oral, 3 capsules/time, tid, for 6weeks;  XELOX | XELOX, 21days/cycle, for 2 cycles  Ox.130 mg/m^2^, ivgtt, 3h, d1  Xe**l** 800mg/m^2^, po, bid, d1-d14 | O1,7 |
| Li YD 2021^[36]^ | RCT | 60/60 | 35-72(47±10.75)/  32-68(46±11.12) | 35/25 | 37/23 | Bifid Triple Viable Capsules, oral, 3 capsules/time, bid,7d;  Supportive and symptomatic management | Supportive and symptomatic management | O1,2,7 |
| Wang JH 2020^[37]^ | RCT | 42/42 | 31-77(45.58±9.25)/ 30-76(45.63±9.65) | 25/17 | 26/16 | Bifid Triple Viable Capsules, oral, 4 capsules/time, tid, for 2weeks; Supportive and symptomatic management | Supportive and symptomatic management | O1,2,7 |
| Zhang YL 2020^[59]^ | RCT | 50/47 | 40-69(52.13±9.89)/ 38-71(53.26±9.54) | 26/24 | 25/22 | Bifid Triple Viable Capsules, oral, 4 capsules/time, tid, for 24weeks; | Capecitabine,0.5g/ time, bid, for 24weeks; | O1,2,3 |
| Zhang YX 2020^[38]^ | RCT | 47/47 | 33-58(45.16±10.52)/ 33-58(45.45±10.40) | 24/23 | 23/24 | Bifid Triple Viable Capsules, oral, 4 capsules/time, tid, for 4weeks;  mFOLFOX | mFOLFOX 14days/cycle, for 2 cycles.Ox. 85 mg/m^2^, ivgtt, 2h, d1,  5-Fu,400mg/m^2^ ivgtt, d1,  this was followed by 5-Fu, 2400 mg/m^2^ for 46h | O1,2,3,7 |
| Liu D 2019^[39]^ | RCT | 47/46 | (48.79±6.31)/  (49.32±5.49) | 27/20 | 25/21 | Bifid Triple Viable Capsules, oral, 3 capsules/time, tid, for 6weeks;  XELOX | XELOX, 21days/cycle, for 2 cycles  Ox.130 mg/m^2^, ivgtt, 3h, d1  Xe**l** 800mg/m^2^, po, bid, d1-d14 | O1,2,5,6,7 |
| Cheng P 2019^[40]^ | RCT | 43/43 | 47-76(60.25±4.11)/  45-79(60.21±4.15) | 25/18 | 24/19 | Bifidobaeterium tetravaccine tablets, 3 capsule/time, bid, for 4weeks;  Supportive and symptomatic management | Supportive and symptomatic management | O1,3 |
| Liu H 2019^[41]^ | RCT | 42/42 | 39-71(57.10±13.50)/  38-72(56.80±12.40) | 25/17 | 26/16 | Bifid Triple Viable Capsules, oral, 3 capsules/time, tid, for 24weeks;  mFOLFOX6 | mFOLFOX6 1 time/14d,2 times/ /cycle, for 6 cycles  Ox. 85 mg/m^2^, ivgtt, 2h, d1,  5-Fu,400mg/m^2^ ivgtt, d1,  this was followed by 5-Fu, 2400 mg/m^2^ for 46h | O1,7 |
| Xu QW 2019^[42]^ | RCT | 30/30 | (61.03±15.28)/  (62.35±13.71) | 20/10 | 18/12 | Bifid Triple Viable Capsules, oral, 3 capsules/time, tid,7d;  12.5% glucose solution | 12.5% glucose solution | O1,2,4 |
| Xie XL 2019^[60]^ | RCT | 66/69 | (62.62±9.627)/  (60.29±9.54) | 44/22 | 43/26 | fructooligosaccharide (25%), xylooligosaccharide (25%), polydex-trose (25%), and resistant dextrin (25%),30g/d,7d;  Supportive and symptomatic management | Supportive and symptomatic management | O3 |
| Yu SF 2018^[43]^ | RCT | 51/51 | (61.78±10.36)/  (62.00±11.28) | 28/23 | 30/21 | Bifidobaeterium tetravaccine tablets, 3 capsule/time, bid,10d;  Enteral nutrition preparations | Enteral nutrition preparations | O1,3,7 |
| Li XW 2018^[44]^ | RCT | 35/35 | (69.20±6.50)/  (68.90±6.40) | 21/14 | 23/12 | Bifid Triple Viable Capsules, oral, 3 capsules/time, tid,12d;  Supportive and symptomatic management | Supportive and symptomatic management | O1,4,7 |
| Zhan YQ 2018^[45]^ | RCT | 41/42 | 40-74(56.23±15.90)/  45-75(55.11±11.67) | 24/17 | 20/22 | Bifid Triple Viable Capsules, oral, 3 capsules/time, tid, for 24weeks;  mFOLFOX6 | mFOLFOX6 1 time/14d,2 times/ /cycle, for 6 cycles.Ox. 85 mg/m^2^, ivgtt, 2h, d1.5-Fu,400mg/m^2^ ivgtt, d1, this was followed by 5-Fu, 2400 mg/m^2^ for 46h | O1,7 |
| Liu F 2017^[46]^ | RCT | 41/41 | (52.10±5.40)/  (51.70±4.80) | 28/13 | 26/15 | Bifid Triple Viable Capsules, oral, 3 capsules/time, bid,28d;  Supportive and symptomatic management | Supportive and symptomatic management | O1,2,5 |
| Sun CY 2017^[47]^ | RCT | 30/30 | 48-74(62.55±5.85)/  46-75(58.45±5.25) | 16/14 | 18/12 | Bifid Triple Viable Capsules, oral, 3 capsules/time, tid,14d;  Supportive and symptomatic management | Supportive and symptomatic management | O1,7 |
| Zuo JD 2017^[48]^ | RCT | 45/45 | 40-80(62.22±8.26)/  40-80(62.17±8.33) | 25/20 | 26/19 | Bifidobacterium bifidum and Lactobacillus rhamnosus triplet, 1 sachet/time, 3 times/d,7d;  Supportive and symptomatic management | Supportive and symptomatic management | O |
| Dai AY 2016^[49]^ | RCT | 39/39 | (67.24±7.15)/  (66.97±6.89) | 23/16 | 25/14 | Bifid Triple Viable Capsules, oral, 3 capsules/time, bid,7d;  Supportive and symptomatic management | Supportive and symptomatic management | O1,2,7 |
| Zhang YJ 2015^[50]^ | RCT | 60/60 | 42-84(66.50±5.70)/  40-80(66.90±5.40) | 33/27 | 34/26 | Bifid Triple Viable Capsules, oral, 3 capsules/time, tid,5d;  Supportive and symptomatic management | Supportive and symptomatic management | O1,2,7 |
| Kotzampassi 2015^[57]^ | RCT | 84/80 | (65.90±11.50)/  (66.40±11.90) | 57/27 | 58/22 | Lactobacillus acidophilus LA-5 1.75x10^9^ CFU, Lactobacillus plantarum 0.5x10^9^ CFU, Bifidobacterium lactis BB-12 1.75x10^9^ CFU and Saccharomyces boulardii 1.5x10^9^ CFU per capsule, 1 sachet/time, 2 times/day,15d | capsules of powdered glucose polymer,1 sachet/time, 2 times/day,15d | O7 |
| AISU 2015^[58]^ | RCT | 75/81 | (68.00±13.80)/  (69.10±11.30) | 47/28 | 44/37 | BIO-THREE tablet contained 2 mg Enterococcus faecalis T110, 10 mg Clostridium butyricum TO-A and 10 mg Bacillus mesentericus TO-A, 6tablets/day,15d; | Supportive and symptomatic management | O7 |
| Zhang JW 2012^[51]^ | RCT | 30/30 | 41-83(66.70)/  39-81(63.00) | 16/14 | 14/16 | Bifid Triple Viable Capsules, oral, 3 capsules/time, tid,5d; Supportive and symptomatic management | Supportive and symptomatic management | O1,2,7 |
| Liu Z 2010^[52]^ | RCT | 50/50 | (65.30±11.00)/  (65.70±9.90) | 28/22 | 31/19 | Lactobacillus plantarum ,cell count＞10^11^ CFU/g, Lactobacillus acidophilus ,LA-11, cell count＞7.0x10^10^ CFU/g and Bifido-bacterium longum ,BL-88, cell count＞5.0x10^10^ CFU/g，2g/d,16d | encapsulated maltodextrin and a 10-g sachet of maltodextrin, 16d | O1 |

**Abbreviations**: **bid**-twice per day; **Bifid Triple Viable Capsules** -210mg/capsule, each capsule contains *Bifidobacterium longum*, *Lactobacillus acidophilus* and *Enterococcus faecalis*, and the number of each live bacteria ＞1.0*10^7^CFU; **Bifidobaeterium tetravaccine tablets** -0.5g/tablet, each tablet contains *Bifidobacterium infantis*, *Lactobacillus acidophilus* and *Enterococcus faecalis* should be not less than 0.5×10^6^CFU respectively; Bacillus cereus should be not less than 0.5×10^5^CFU; **C**-control group; **Clostridium butyricum Live Tablets** -350 mg/tablet, the number of live bacteria per tablet＞1.5×10^7^ CFU/g; **Compound probiotics** **-** Each bag contains oligofructose (40%), inulin (50%), *Bifidobacterium* *youth*, *Bifidobacterium bifidum*, *Lactobacillus acidophilus*, *Lactobacillus casei*, *Lactobacillus rhamnosus* and *Lactobacillus plantarum*, with a total live count of 10 billion CFU/bag**; d**-day; **ID** -intravenous drip; **ivgtt** -injection venosa gutta; **M**- Microecological preparation group; **n**-number;**O**- outcomes, **O1**- Intestinal flora related indicators ; **O2**- intestinal mucosal barrier function related indicators ;**O3**- immune function related indicators;**O4**- Inflammatory factor related indicators ;**O5**- Clinical effectiveness ; **O6**-tumor markers and related factors ;**O7**- Adverse reactions ; **Ox.**-oxaliplatin; **qd**-once per day; **tid**-thrice per day; **Xel/Cap.**-capecitabine; **XOS**-Xylose oligomers; **XELOX**-Ox. + capecitabine
